# Leveraging clinical data across healthcare institutions for continual learning of predictive risk models

**DOI:** 10.1038/s41598-022-12497-7

**Published:** 2022-05-19

**Authors:** Fatemeh Amrollahi, Supreeth P. Shashikumar, Andre L. Holder, Shamim Nemati

**Affiliations:** 1 Division of Biomedical Informatics, University of California San Diego, San Diego, USA; 2 Division of Pulmonary, Critical Care, Allergy and Sleep Medicine, Emory University School of Medicine, Atlanta, USA

**Keywords:** Infectious diseases, Computer science

## Abstract

The inherent flexibility of machine learning-based clinical predictive models to learn from episodes of patient care at a new institution (site-specific training) comes at the cost of performance degradation when applied to external patient cohorts. To exploit the full potential of cross-institutional clinical big data, machine learning systems must gain the ability to transfer their knowledge across institutional boundaries and learn from new episodes of patient care without forgetting previously learned patterns. In this work, we developed a privacy-preserving learning algorithm named WUPERR (Weight Uncertainty Propagation and Episodic Representation Replay) and validated the algorithm in the context of early prediction of sepsis using data from over 104,000 patients across four distinct healthcare systems. We tested the hypothesis, that the proposed continual learning algorithm can maintain higher predictive performance than competing methods on previous cohorts once it has been trained on a new patient cohort. In the sepsis prediction task, after incremental training of a deep learning model across four hospital systems (namely hospitals H-A, H-B, H-C, and H-D), WUPERR maintained the highest positive predictive value across the first three hospitals compared to a baseline transfer learning approach (H-A: *39.27%* vs. *31.27%*, H-B: *25.34%* vs. *22.34%*, H-C: *30.33*% vs. *28.33%*). The proposed approach has the potential to construct more generalizable models that can learn from cross-institutional clinical big data in a privacy-preserving manner.

## Introduction

The remarkable resurgence of artificial intelligence and its impact on industrial automation, optimization of customer satisfaction and revenue over the past decade has resulted in a growing interest in the application of related technologies to healthcare^1–3^. In particular, deep learning techniques have gained increased attention in clinical medicine, including screening and triage, diagnosis, prognostication, decision support and treatment recommendation^4–13^. To gain wide clinical adoption, deep learning-based clinical models have to be generalizable and portable, and ensure the privacy of patients whose data are used for model training and evaluations^14,15^. In practice, models trained on data from a single healthcare system often suffer from lack of generalizability due to differences in local demographics, laboratory equipment and assays, electronic health records (EHR), frequency of data measurement, and variations in clinical and administrative practices including coding and definitions of various clinical diagnoses^16^. It has been argued that clinical big data when combined with the inherent flexibility of deep learning models to learn from new data/experiences could in theory address some of these heterogeneity. However, healthcare data remains siloed and data accessibility and patient privacy pose a substantial challenge to fully leveraging the power of advanced analytics in the healthcare domain^15,17^. As such, in the present day, typical clinical data utilized for model development are often several orders of magnitude smaller than those fueling the industrial applications of deep learning^18^.

A recent independent and external validation of a widely used machine learning-based sepsis prediction risk score highlighted the issue of model generalizability in the presence of data distribution shift and changes in the population case-mix^19,20^. A potential solution to improving external validity of deep learning systems is to fine-tune such models in every new care setting (aka, Transfer Learning)^21,22^. However, this approach may result in many versions of the same algorithm operating in different care settings, which raises regulatory concerns regarding change-management and scientific challenges regarding the production of generalizable knowledge^23^. Therefore, it is desirable to design learning algorithms and models that can leverage patient data across diverse cohorts of patients in a privacy-preserving manner and with well-defined change control plans^24^ that can maintain acceptable performance while managing potential risk to patients.

Federated and/or distributed learning is a method of learning models from data distributed across different sources^25^. Privacy-preserving methods have been proposed to leverage such data for learning while respecting institutional boundaries and autonomy over patient data^26,27^. Such models assume that data is available at once across multiple sites^26,28^, however, in practice deep learning models are often developed and rolled out over time in a sequential manner (e.g., as a business expands its customer-base), where a model trained and validated on data from a single healthcare institution (Hospital-A) is disseminated and implemented at a second (Hospital-B) and subsequent sites (Hospital-C, etc.). As an alternative to the two extremes of (1) maintaining all model coefficients fixed, and (2) site-specific model deployment where the model coefficients are fine-tuned to every local population of patients, one can imagine a scenario where a single model continues to learn from new cohorts of patients and maintains generalizability. This scenario is closely related to the continual learning (aka, lifelong learning) framework in the deep learning literature, where a model is trained to learn a series of tasks sequentially (e.g., predicting mortality in Hospital A, B, C, etc.) while maintaining acceptable performance on prior tasks (aka, overcoming ‘catastrophic forgetting’)^29–31^.

**Figure 1:**
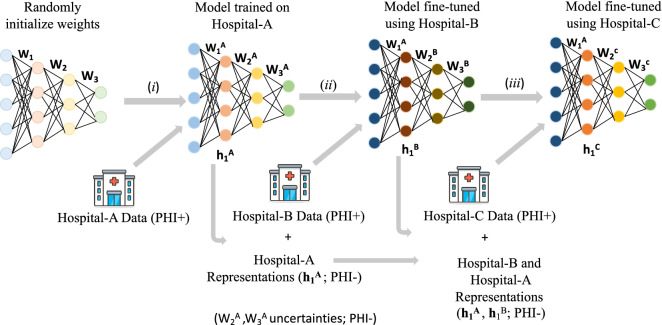
Schematic diagram of the WUPERR algorithm. The training starts with a randomly initialized set of weights, which are trained on the first task (e.g., prediction on Hospital-A data). In all subsequent learning tasks the input layer weights ($W_1^A$) are kept frozen. The optimal network parameters, the parameter uncertainties under task-A, and the set of representations from training cohort of Hospital-A ($\{h_1^A\}$) are then transferred to Hospital-B. The deeper layers of the model are fine-tuned to perform the second task (e.g., prediction on Hospital-B data) through replaying the representation of Hospital-A and Hospital-B data. Similarly, the optimal parameters and their uncertainty levels along with the Hospital-A and Hospital-B representations are transferred to Hospital-C to fine-tune the model on performing the third task. Note, at no time protected health information (PHI+) leaves the institutional boundaries of a given hospital. Finally, at the time of evaluation (on testing data) at a given task, the model is evaluated on all the hospital cohorts.

Despite the need for robust continual learning algorithms in clinical settings, applications of such methods to clinical predictive modeling remain scarce^32^. Here we consider a clinically significant problem involving prediction of sepsis in critically ill patients. Using data across four sepsis cohorts, we developed and validated a continual learning framework (see Fig. 1) for sequentially training predictive models that maintain clinically acceptable performance across all cohorts while preserving patient data privacy. Drawing inspiration from the latest developments in the lifelong learning literature, we propose a joint elastic weight consolidation (EWC)^33^ and episodic representation replay (ERR)^34–37^ framework to continuously update our predictive models on new patient cohorts. Figure 1 illustrates the basic building blocks of the proposed weight uncertainty and episode representation replay (WUPERR) framework. WUPERR achieves continuous learning through two mechanisms: (1) tracking network weights that are essential to prior tasks and thus should remain unchanged over the course of learning a new task; and (2) interleaving training data representations from prior tasks during acquisition of a new task. To achieve privacy, WUPERR replaces raw patient-level features with hidden representations learned via a neural network (e.g., activation of neurons in the first layer of the network), thus obviating the need for moving protected health information outside institutional boundaries.

The aim of this study was to examine whether the proposed continuous learning approach provides improved generalizability across all patient cohorts. We hypothesized that incorporation of EWC and ERR methodologies would result in a more generalizable model than a Transfer Learning approach previously explored in this context^21^. To further explore the effect of continuous learning on various network parameters we conducted layer-wise analysis of weight adaptation with learning of new tasks. We tested the WUPERR algorithm in the context of sequential training of a deep learning model for early prediction of sepsis across four geographically distinct populations within the United States (total of 104,322 patients). Our proposed continual learning approach allows for leveraging data across institutional boundaries to sequentially train generalizable predictive risk scores in a privacy-preserving manner.

## Results

We evaluated the performance of the proposed learning algorithm for early prediction of onset of sepsis in hospitalized patients across four healthcare systems. A comparative study of WUPERR against several baseline models is shown in Supplementary material Figs. S4–S6, however, for the sake of brevity we only report the performance of WUPERR against transfer learning in the next section.

**Figure 2:**
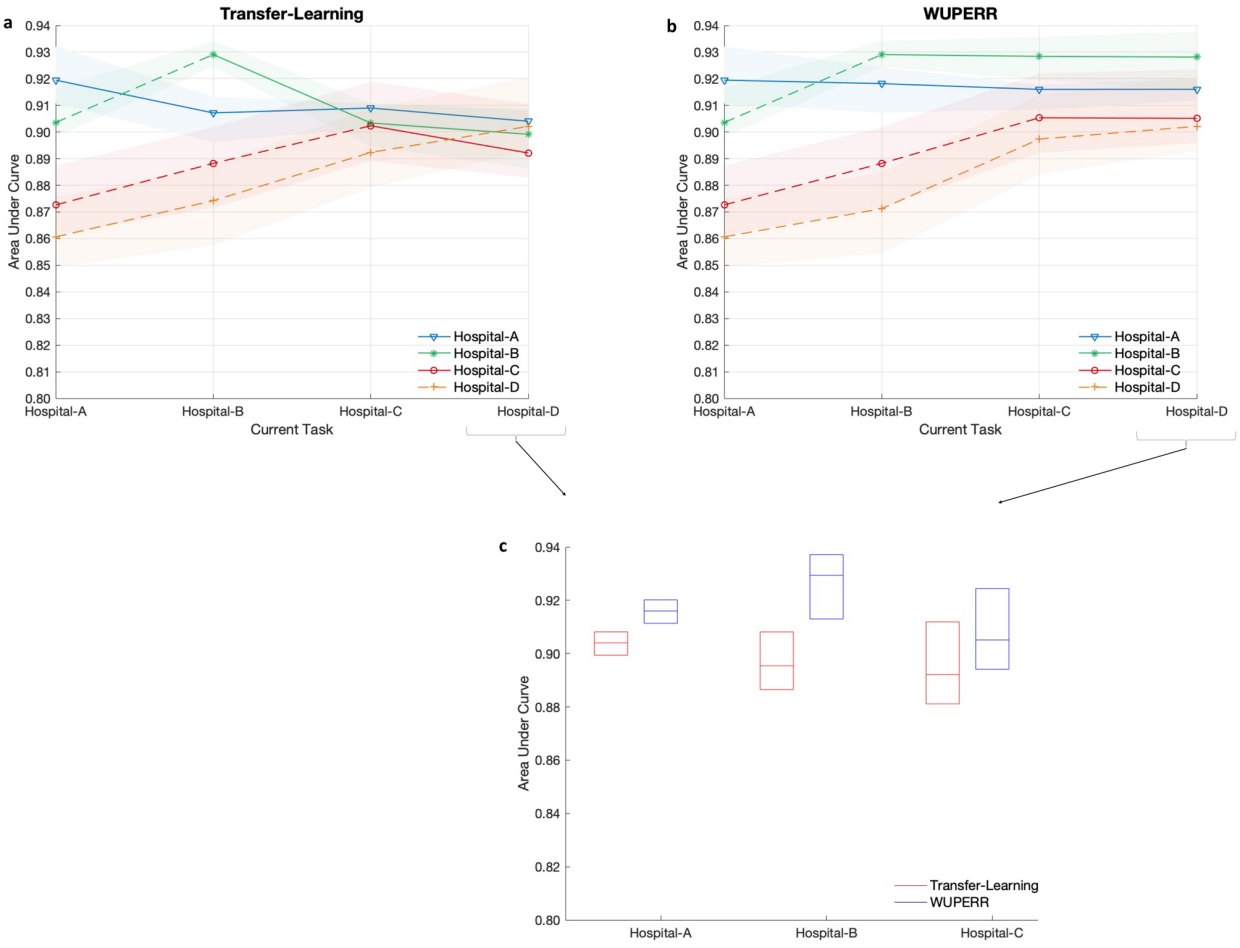
Evaluation of continual learning models for early predicting of onset of Sepsis, measured using Area Under the Curve (AUC) metric. (**a**) Illustrates AUC of a model (median[IQR]) trained using transfer learning. The model performance is reported (using different markers; see legend) across all the cohorts after sequential training on data from a given hospital on the x-axis. (**b**) shows the AUC of the proposed WUPERR model, under the same experimental set-up as (**a**). At the time of evaluation (on testing data) at a given site, the model is evaluated on all the hospital cohorts. The solid line-style indicates that at the time of model evaluation (on testing data) at a given site, the model had already seen the training data from that site. For instance, since the model is first trained on Hospital-A data, the performance of the model on this dataset after continual learning on all subsequent hospitals is shown in solid line-style to signify that the model had already seen this patient cohort in the past. (**c**) summarizes the model performance (median[IQR]) on Hospitals A–C after continual learning on all four hospitals with Transfer learning (red) and WUPERR (blue).

**Figure 3:**
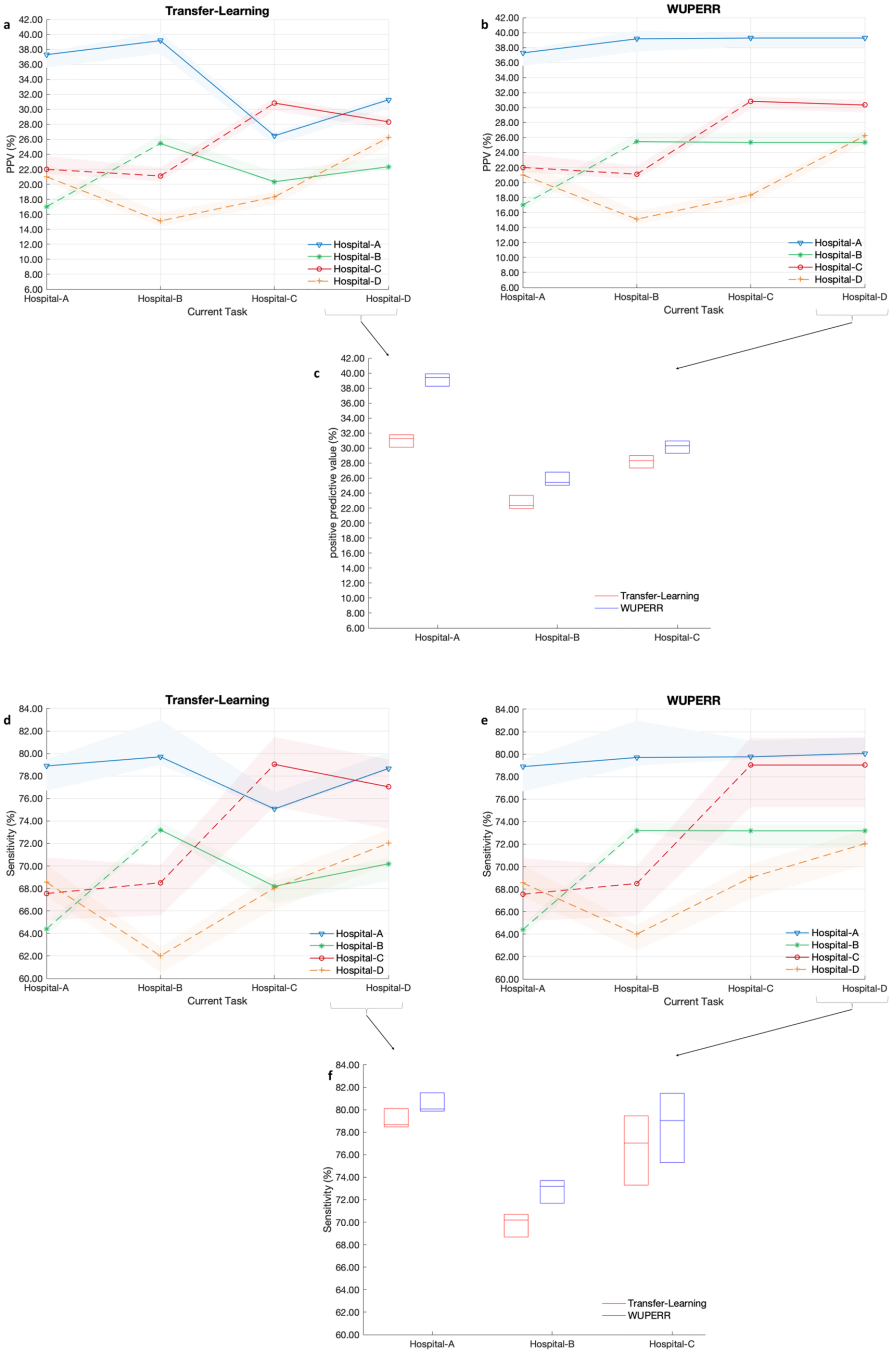
Evaluation of continual learning models for early predicting of onset of Sepsis, measured using positive predictive value (PPV) and sensitivity. (Atlanta) Illustrates the PPV of a model (median[IQR]) trained using transfer learning (measured at fixed threshold of 0.41 corresponding to 80% sensitivity at Hospital-A after Task 1, for all folds and across all tasks). The model performance is reported (using different markers; see legend) across all the cohorts after sequential training on data from a given hospital on the x-axis. (Atlanta) shows the PPV of the proposed WUPERR model, under the same experimental set-up as (Atlanta). (Atlanta) summarizes the model performance (median[IQR]) on Hospitals A-C after continual learning on all four hospitals with Transfer learning (red) and WUPERR (blue). (**d**–**f**) summarize the model sensitivity results under the same experimental protocol.

### Sepsis prediction setting

The WUPERR framework was used to train a model to sequentially predict the onset of sepsis (defined according to the Sepsis-3 consensus definitions for Sepsis and Septic Shock) four hours in advance^38^. To investigate the impact of variations in data distributions on our model performance, we trained our model sequentially on over 104,000 patients belonging to four critical care centers with various underlying demographic characteristics. The model was first trained on the Hospital-A dataset (Task 1), followed by Hospital-B (Task 2), Hospital-C (Task 3) and Hospital-D (Task 4). The performance of the model sequentially trained using the WUPERR framework was compared with a baseline transfer learning approach. Figure 2a–c, show the performance of WUPERR on the four hospital datasets, where the model was trained on one cohort at a time and the performance is reported on testing data from all other cohorts (previous and subsequent cohorts). With the transfer learning approach, we observed that with the progression in training on new cohorts the model performance degenerated on previous cohorts. Whereas sequential training by WUPERR enabled the model to maintain comparable performance on older tasks. For example, at the end of Task 4 with transfer learning, AUC of the model on Task 2 was 0.90 [0.89–0.91], a drop from the AUC of 0.93 [0.92–0.94] when the model was trained on the data from Hospital-B (corresponding to task 2). In comparison, at the end of Task 4 with WUPERR, the model maintained its performance on Task 2 with an AUC of 0.93 [0.91–0.94]. Notably, we observed that the superiority of WUPERR over transfer learning grow as the number of subsequent training cohorts the model was exposed to increased (see Fig. 2c, performance on Hospital-A at the end of training on hospital-D). Additionally, we observed that at the end of Task 4, the model trained with the WUPERR approach performed superior to transfer learning across all the Hospital cohorts (see Fig. 2b).

In Fig. 3 we compared the positive predictive value (PPV) of the model sequentially trained on four cohorts using the WUPERR approach versus the baseline transfer learning approach. A decision threshold corresponding to 80% sensitivity was chosen after completion of training on Task 1. This decision threshold was then used to measure positive predictive value (PPV) for all the remaining tasks. We observed that WUPERR consistently outperformed the transfer learning approach across all the tasks (see Fig. 3a–c). For instance, with WUPERR the positive predictive value (PPV) for Hospital-A improved from 37.28 [35.57–37.69] after Task 1 to 39.27 [38.11–39.78] by the end of Task 4, whereas with transfer learning approach the positive predictive value (PPV) dropped to 31.28 [30.11–31.78] by the end of Task 4. Additionally, WUPERR was able to maintain consistent sensitivity levels on the Hospital-A cohort while being sequentially trained on Tasks 2, 3, and 4 (79.70 [78.50–82.57], 79.76 [79.57–81.20], 80.06 [79.87–81.50], respectively). In comparison, the sensitivity level on the Hospital-A cohort dropped below 80% when the model was trained on Tasks 2, 3 and 4 in the case of transfer learning approach (see Fig. 3d). Similar patterns for sensitivity were observed for the other hospital cohorts. Finally, we observed that WUPERR was robust to the training order and consistently outperformed the transfer learning approach even when the ordering of hospitals was swapped (see Supplementary Figs. S7–S12).

## Discussion

In this study we designed and validated a continual learning algorithm for training generalizable clinical predictive analytics models across multiple patient cohorts. WUPERR integrates rehearsal memory with weight uncertainty propagation, and enables clinical deep learning models to learn new tasks while maintaining acceptable performance across prior tasks. We evaluated our proposed algorithm on four consecutive tasks involving early prediction of sepsis in hospitalized patients. Our results indicate that WUPERR can successfully deal with data distribution shifts that often adversely affect the generalizability of clinical predictive models. By the virtue of using data representations for continual learning, WUPERR allows the raw training data to remain at each site and therefore maintains privacy and autonomy of healthcare data. We compared WUPERR against several baselines, including Transfer Learning^21^, EWC^33^, and Experience Replay using three clinically relevant performance metrics, namely AUCroc, Positive Predictive Value, and Sensitivity. One may expect that learning a site-specific model should achieve the best performance, although such a model may not generalize well to external sites. WUPERR outperformed baseline Transfer Learning and EWC in terms of all three metrics to alleviate forgetting. One of the main advantages of WUPERR is the ability to learn from embedded representation of data points which makes WUPERR an appropriate approach for privacy-preserving continual learning.

Research on machine learning and deep learning has produced promising results in identification, diagnosis, and delivery of treatments in healthcare^39,40^. Improved performance of deep learning algorithms comes at the cost of requiring large and diverse datasets^41^. However, patient privacy and data governance considerations have contributed to data silos and have made the task of constructing large multicenter datasets impractical. Some of the challenges of learning complex models from data silos have been addressed by Federated learning, where a decentralized learning algorithm relies on local model updates to construct a global model^25,42,43^. Huang et al., introduced the community based federated learning (CBFL) framework to predict prolonged ICU stay and mortality^44^. Qayyum et al., used clustered federated learning (CFL) for identifying patients with Covid-19^45^. While promising, federated learning models tend to learn an average model that may perform suboptimally within any given local site. In particular, standard federated learning methods do not address the problem of data distribution shift and model drift that result from differences in patient demographics and workflow-related practices. On the other hand, continual learning methods (such as WUPPER) allow models to incrementally learn new tasks while preserving their performance on prior tasks. This allows a model to adapt to dynamic changes and shifts in data distribution across different healthcare sites. A recent longitudinal analysis of a sepsis alert algorithm across four geographically diverse health systems reported significant dataset shift due to a change in the case-mix over time^46^. As such, algorithm monitoring^47^ and continual learning are needed to ensure such systems adapt to the underlying changes in data distribution and can maintain a high level of accuracy.

This study has several limitations. The proposed learning method allows a model to adapt to shifting data distributions across clinical sites, however, a key requirement is the quality of input data and labels. Recently, conformal prediction was introduced to provide a probabilistic framework for assessing out-of-distribution samples and to detect outliers and noisy data^47^. WUPERR can be used in association with conformal prediction to control the quality of input data at each site for continual learning. In addition, differences in quality of labels at various sites can pose a challenge to continual learning. Combining WUPERR with methods for assessing and correcting label noise may provide a mechanism for training high-quality models. Moreover, WUPERR does not address the problem of partial data availability, but recent work in continually growing neural networks can be combined with WUPERR to design algorithms that can leverage additional variables and features in new datasets^48,49^. Finally, the datasets used in this study were collected from major academic medical centers and may not be representative of smaller community and rural hospitals. However, our proposed framework is likely to benefit smaller hospitals that may not have the necessary resources to maintain large clinical data warehouses, since fine-tuned pre-trained neural networks have been shown to outperform neural networks trained from scratch on smaller datasets^22^. In summary, our findings provide significant clinical evidence for the applicability of continual learning to design and update of generalizable clinical predictive models.

## Methods

### Study population

A total of 104,000 adult patients admitted to the ICUs at four geographically diverse healthcare institutions, including UC San Diego Health, Emory University Hospital, Grady Hospital, and the Beth Israel Deaconess Medical Center (henceforth, Hospital-A, Hospital-B, Hospital-C and Hospital-D, respectively) made up the study cohort. All analyses were performed in accordance with relevant guidelines and regulations. The use of de-identified data utilized in this study was approved by the Institutional Review Board (IRB) of UC San Diego (IRB$\#$191098), the IRB of Emory University/Grady Hospital (IRB$\#$110675), and the Beth Israel Deaconess Medical Center (IRB$\#$0403000206)^50^ and the requirement for informed consent were waived by the IRB committees of UC San Diego, Emory University/Grady Hospital, and the Beth Israel Deaconess Medical Center, as the use of de-identified retrospective data does not require patient consent under the Health Insurance Portability and Accountability Act (HIPAA) privacy regulations. Patients 18 years or older were followed throughout their ICU stay until time of first episode of sepsis or otherwise time of transfer out of ICU. We followed the latest guidelines provided by the Third International Consensus Definitions for Sepsis (Sepsis-3)^38,51^ which defined sepsis as a life-threatening organ dysfunction caused by a dysregulated host response to infection. As such, the two main criteria for establishing onset time of sepsis included: (1) evidence of acute organ dysfunction, and (2) suspicion of infection. Clinical suspicion of infection was defined by blood culture draw and new start of intravenous (IV) antibiotics continued for > = 3 consecutive days (excluding prophylactic use) satisfying either of the following conditions: (a) if a blood culture draw was ordered first, then antibiotics order had to occur within the following 72 h, or (b) if antibiotics order occurred first, then a blood culture draw had to occur within the next 24 hours. Evidence of organ dysfunction was defined as an increase in the Sequential Organ Failure Assessment (SOFA) score by two or more points. In particular, evidence of organ dysfunction occurring 48 h before to 24 h after the time of suspected infection was considered, as suggested in Singer et al.^51^. Finally, the time of onset of sepsis was taken as the time of clinical suspicion of infection. To allow for initial examination and stabilization of patients and adequate data collection for prediction purposes, we focused on sequential hourly prediction of sepsis starting at hour four after ICU admission. Patients who were identified as having sepsis prior to prediction start time or those with no measurement of heart rate or blood pressure prior to the prediction start time or those whose length of stay within a given care unit were more than 21 days were excluded.

### Data preparation

A total of 40 clinical variables were extracted across the four hospitals (see Supplementary materials Fig. S2). Additionally, for every vital signs and laboratory variable, their local trends (slope of change) and the time since the variable was last measured (TSLM) were recorded, resulting in a total of 108 features (the same set of variables have been used in a previously published study^47^). The patient characteristics of all the four cohorts have been tabulated in Supplementary Table S1. All continuous variables are reported as medians with 25% and 75% interquartile ranges (IQRs). Binary variables are reported as percentages. All vital signs and laboratory variables were organized into 1-h and 1-day non-overlapping time series bins to accommodate for different sampling frequencies of available data for the sepsis cohort. All the variables with sampling frequencies higher than once every hour (or day) were uniformly resampled into 1-h (or 1-day) time bins, by taking the median values if multiple measurements were available. Variables were updated hourly when new data became available; otherwise, the old values were kept (sample-and-hold interpolation). Mean imputation was used to replace all remaining missing values (mainly at the start of each record).

### Development of WUPERR

WUPERR combines Episodic Representation Replay (ERR) and Weight Uncertainty Propagation (WUP) to enable continual learning of tasks while mitigating the problem of catastrophic forgetting. The goal of WUPERR is to minimize the drop in performance on older tasks when the model is trained on a new task (i.e., a new hospital). WUPERR attempts to achieve this goal through consolidation of network parameters important to model prediction on prior tasks (via a targeted weight regularization scheme) and episodic experience replay (by maintaining sample data representations encountered during prior training and periodically revisiting those examples during re-training). Figure 1 shows the schematic diagram of the WUPERR algorithm.

Let *N*, *J*, *K* be the number of parameters of the neural network, the number of training epochs, and the total number of tasks, respectively. At training time of task *k*, the loss $L(j;\theta )$ calculated at epoch *j* is as follows:

1 $$\begin{aligned} L(j,\theta )=L_{CE}(j;\theta )+\frac{\gamma }{2}\sum _{n=1}^{N}I_{n}^{k}(j-1)(\theta _{n}^{k}(j-1)-\theta _{n}^{k-1})^2 \end{aligned}$$

where $L_{CE}(j;\theta )$ corresponds to the cross-entropy classification loss, $\theta _{n}^{k}(j-1)$ corresponds to the n-th parameter of the neural network from the previous epoch, $I_{n}^{k}(j-1)$ is an approximation of Fisher information (inverse of uncertainty) associated with parameter $\theta _{n}$ during task *k* and epoch $j-1$. The approximate Fisher information corresponding to parameter $\theta _{n}$ during task *k* and epoch *j* is computed as follows:

2 $$\begin{aligned} I_{n}^{k}(j)=\beta *I_{n}^{k}(j-1)+(1-\beta )\left( \frac{\partial L(j;\theta )}{\partial \theta _n^k}\right) ^2 \end{aligned}$$

Note that the magnitude of the gradient corresponds to the degree of steepness of the loss surface around a point in the parameter space, which in-turn provides a measure of information gain. For task $k (k = 2,\ldots , K)$, $I_n^k$ is initialized as $max (I_n^1,\ldots , I_n^{k-1})$.

We used Bayesian Optimization to set the cost function regularization parameter (Eq. (1)) and uncertainty estimation moving average parameter (Eq. (2)), which resulted in the optimal values of $\gamma =0.99$ and $\beta =0.80$, respectively.

Note that, after task 1, parameters corresponding to the first layer of the neural network are frozen. Additionally, after completion of training on each Task *k*, the hidden representations ($h_1^k$; output from the first layer of neural network) corresponding to a random sample of patients from Hospital-k are stored. From Task 2 onwards, we fine-tune the neural network (except for the first layer) with data from the new patient cohort (Hospital-k) and hidden representations stored from previous tasks. Note that, empirically, freezing of the layer-1 weights had negligible impact on model performance since model re-training predominantly affects the upper layer parameters (see Supplementary Fig. S13).

### Baseline models

The performance of the WUPERR algorithm was compared against four baseline models, listed below:

- *Site-specific training*: In this approach, we trained the model in isolation at each hospital site wherein a new model is trained on each task independently.
- *Transfer learning*: Transfer learning assumes that the source and target tasks are derived from the same feature space, as a result of which transferring knowledge from prior tasks might accelerate the learning procedure on new tasks and thereby improve model performance. In this approach, parameters of the neural network after training on task k-1, were transferred over to task k and were further fine-tuned using data from task k.
- *Transfer learning-freeze*: In this approach, the first layer of the neural network was frozen after training on task 1. Parameters of the neural network after training on task k-1, were transferred over to task k and were further fine-tuned (all layers except the first layer) using data from task k.
- *Elastic weight consolidation (EWC)*^33^: This approach relies on regularization terms to avoid forgetting. EWC protects the neural network performance on old tasks by slowing down the learning process on selected weights and staying in a region corresponding to lower error for prior tasks while learning a new task. To identify weights that carry more information, EWC relies on a fisher information matrix. EWC implements the sum of quadratic penalties over already seen tasks to avoid forgetting in DNNs.
- *Episodic representation reply (ERR)*: In ERR, we use representations of data from previous tasks in addition to data from the current to fine tune a model. Supplementary Fig. S13 shows the layer-wise Frobenius norm of changes in our network weights, as training continued from Task-1 through Task-4. We observed the greatest changes in the network weights at the deeper layers, which may suggest that these layers are more important to learning a new task. consequently, it was observed that freezing the weights within the first network layer had little effect on the ability of the network to adapt to a new dataset. This enabled us to use the first layer (after training on Task 1) as an encoding network to obtain representations for the upper network layers. From Task 2 onwards, we used these input data representations at every new site, in conjunction with the representation of data from prior sites, to train the model. The latter (i.e., replaying data representations from prior tasks) enabled the network to remember the older tasks while learning from a new dataset.

### Hyperparameters

The prediction model was a four-layer (two hidden layers) fully connected neural network, with rectified linear unit (ReLU) activation functions. For training, the Adam optimizer with a learning rate of 1e-3 was employed. The various network architecture parameters and hyperparameters have been listed in Supplementary Table S2. Bayesian optimization was performed (using the development cohort of Task 1) to obtain the optimal hyperparameters.

### Training and evaluation

At each site, we split the task dataset 80–20% for training and model testing, respectively. Within each iteration of training we combined the new task data representations (i.e., training data outputs from the first network layer) with randomly selected data representations from prior tasks. Across all of the four datasets, tenfold cross-validation was used for training and testing purposes. In the sepsis cohort, the Hospital-A training set was standardized by first applying normalization transformations, followed by subtracting the mean and dividing by the standard deviation. Next, all remaining datasets in the sepsis cohort (Hospitals B, C and D) were normalized using exactly the same transformations utilized in the training data.

WUPERR was compared with several baseline continual learning methods to predict sepsis across four hospitals on three metrics including AUCroc, positive predictive value and sensitivity. Since the sepsis incidence rates varied across the different health care sites, we also report the model performance using the positive predictive value metric, at a threshold corresponding to the sensitivity of 80% on task 1. Additionally, at the time of evaluation (on testing data) at a given site, the model was evaluated on all the hospital cohorts. It is to be noted that a solid line-style (in Figs. 2, 3 and Supplementary material Figs. S4–S12) is used to represent a hospital site whose training data has already been seen by the model whereas a dashed-line indicates that the model has not been trained on the corresponding hospital site yet. For instance, in Fig. 2, since the model is first trained on Hospital-A data, the performance of the model on this dataset after continual learning on all subsequent hospitals is shown in solid line-style to signify that the model had already seen this patient cohort in the past. Data preprocessing was performed using Numpy^52^ and The models were implemented using using TensorFlow^53^.

## Supplementary Information

Supplementary Information.

## Data Availability

Sample datasets analyzed in the current study are available via PhysioNet Challenge 2019 website (https://physionet.org/content/challenge2019/) and the WUPERR_CLP repository (https://github.com/NematiLab/WUPERR_CLP). For more information, please contact the corresponding author.

## Code availability

The code used to train and validate the model will be made available at https://github.com/NematiLab/WUPERR_CLP.
