# Supplementary material for: Leveraging clinical data across healthcare institutions for continual learning of predictive risk models
Source: Sci Rep. 2022 May 19;12:8380. doi: 10.1038/s41598-022-12497-7 (PMC9117839; doi:10.1038/s41598-022-12497-7)
Supplement: Supplementary file 1 — Supplementary Information. [file 41598_2022_12497_MOESM1_ESM.pdf]

Supplementary Material for "Leveraging Clinical Data Across Healthcare Institutions for Continual Learning of Predictive Risk Models"

Fatemeh Amrollahi, MS[1], Supreeth P. Shashikumar, PhD[1], Andre L. Holder, MD[2], Shamim Nemati, PhD[1*]

[1]Division of Biomedical Informatics, University of California San Diego, San Diego, USA

[2]Division of Pulmonary, Critical Care, Allergy and Sleep Medicine, Emory University School of Medicine, Atlanta, GA

[*]To whom correspondence should be addressed; E-mail: snemati@health.ucsd.edu

Supplementary Note: Patient characteristics

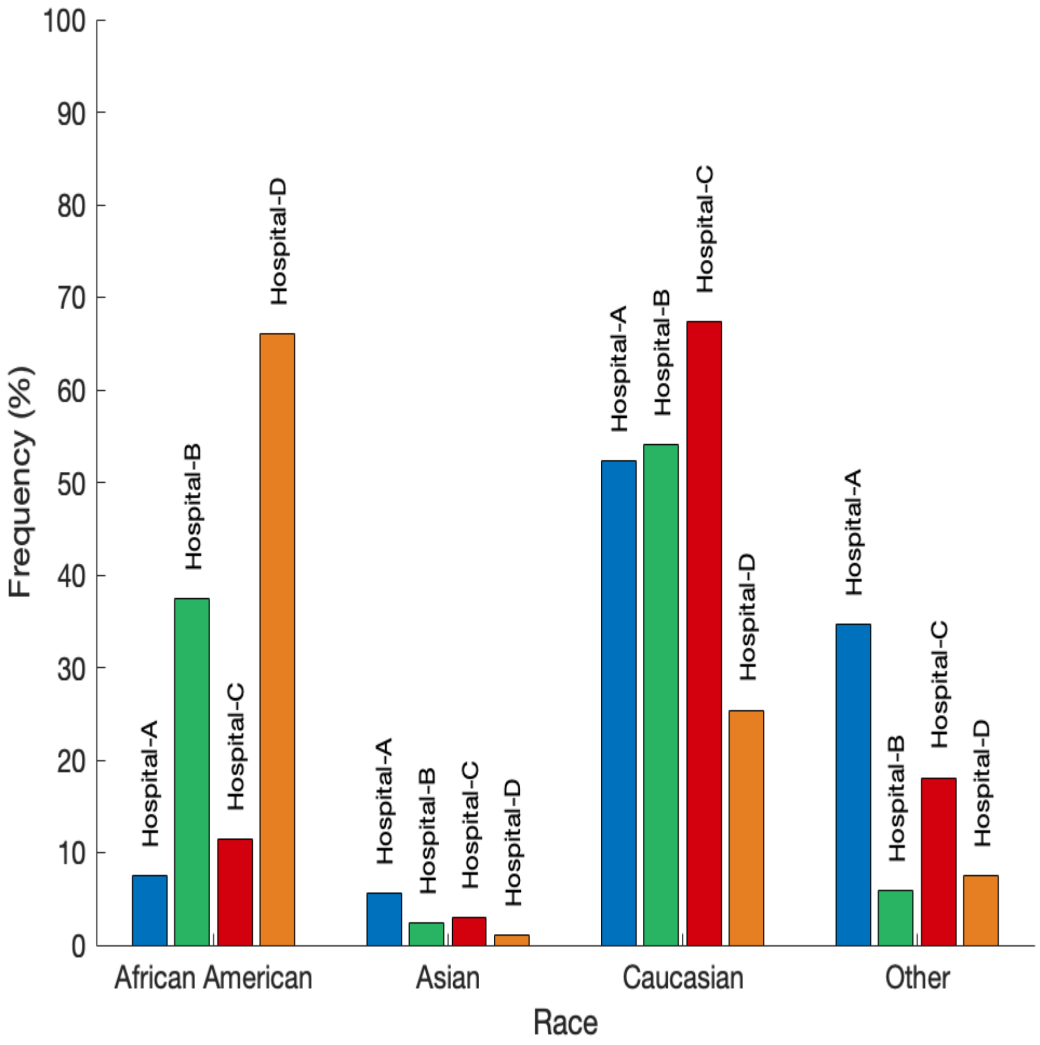

Supplementary Figure S1: Distribution of race/ethnicity per cohort

Supplementary Table S 1: Summary of patient characteristics of the four cohorts considered in this study (including University of California San Diego Health, Emory University Hospital, MIMIC IV, and Atlanta's Grady Hospital, referred to as Hospital-A, Hospital-B, Hospital-C, and Hospital-D respectively)

	Hospital-A		Hospital-B		Hospital-C		Hospital-D	
Dataset	Non-Septic	Septic	Non-Septic	Septic	Non-Septic	Septic	Non-Septic	Septic
Patients (#)	13,208	3,825	37,899	7,913	28,892	7,916	2,999	992
Male, n(%)	7,89(60.4%)	2,463 (64.4%)	20,313(53.6%)	4,305(54.4%)	16,000(55%)	4,439(56%)	2,303(65%)	779(68%)
Race (%)								
Caucasian	7,013(53.1%)	1,897(49.6%)	20,882(55.1%)	3,885(49.1%)	19540(67.7%)	5,288(66.8%)	789(26.3%)	227(22.9%)
African American	977(7.4%)	295(7.7%)	13,871(36.6%)	3,331(42.1%)	3340(11.5%)	843(10.6%)	1,946(64.9%)	688 (69.4%)
Asian	713(5.4%)	241(6.3%)	947(2.5%)	166(2.1%)	896(3.1%)	221(2.8%)	36(1.2%)	7(0.7%)
Other	4,505(34.1%)	1,410(36.8%)	2,199(5.8%)	531(6.7%)	5,116(17.7%)	1564(19.7%)	228(7.6%)	70(7.0%)
Age(yrs), median[IQR]	60.2[46.8 71.3]	60.7[48.1 72.2]	62[50 72]	62[50 72]	64[52 76]	64[52 75]	55[37 66]	57[42 67]
T_sepsis (hrs), median[IQR]	-	22.6[11.2 52.6]	-	26.3 [10.3 67.3]	-	9[1 36]	-	37[12 85]
ICU-LOS(hrs), median[IQR]	45.6 [25.8 80.9]	188.7[93.4 381.3]	45.2[25.5 76.5]	176.3[87.2 353.1]	36[60 24]	95[49 201]	56.8[32.0 94.4]	256.7 [127.3 449.1]
SOFA	3[1 5]	7[4 10]	2[1 5]	7[4 10]	2[1 6]	6[3-9]	3[1 6]	9[6 12]
Mortality n(%)	357(2.7%)	845 (22.1%)	827 (2.3%)	1,282(16.2%)	2059(7.1%)	1546(19.5%)	214(6.0%n)	233(20%)

Supplementary Note: Model parameters

Supplementary Table S2: Hyperparameters of WUPERR

Parameter	Value
N Hidden Layers	2
N hidden units	[40,25]
N Input features	153
Mini-Batch (Septic incidence)	5000 (50%)
Learning rate	1e-3
L1 regularizer	1e-3
L2 regularizer	1e-2
γ	0.99
β	0.8

Supplementary Note: List of clinical variables

Variable	Measurement Unit	Variable	Measurement Unit
Vital Signs (Dynamical Features)			
Heart rate	*beats/minute*	Mean Arterial Pressure	*mmHg*
Pulse oximetry	*%*	Diastolic BP	*mmHg*
Temperature	*degC*	Respiration rate	*breaths per minute*
Systolic BP	*mmHg*	End tidal CO_2	*mmHg*
Laboratory values (Dynamical Features)			
Excess bicarbonate	*mmol/L*	Serum Glucose	*mg/dL*
Bicarbonate	*mmol/L*	Lactic acid	*md/dL*
Fraction of inspired Oxygen	*%*	Magnesium	*mmol/dL*
pH	-	Phosphate	*mg/dL*
Partial pressure of CO_2 from arterial blood	*mmHg*	Potassium	*mmol/L*
Oxygen saturation from arterial blood	*%*	Total Bilirubin	*mg/dL*
Aspartate transaminase	*IU/L*	Troponin I	*ng/mL*
Blood Urea Nitrogen	*mg/dL*	Hematocrit	*%*
Alkaline phosphate	*IU/L*	Hemoglobin	*g/dL*
Calcium	*mg/dL*	Partial Thromboplastin Time	*seconds*
Chloride	*mmol/L*	White Blood Cell count	*count*10^3/μL*
Creatinine	*mg/dL*	Fibrinogen	*mg/dL*
Bilirubin direct	*mg/dL*	Platelets	*count*10^3/μL*
Demographics			
Age	*Years*	Hours between hospital admit and care unit admit	*hours*
Gender	*Male/Female*	Duration until current time	*hours*
Care Units	*Medical/Surgical ICU unit*	--	--

Supplementary Figure S2: List of clinical variables

Supplementary Note: Percentage of Variable Missingness

Supplementary Table S3 tabulated the percentage of the missingness of variables across the 4 hospitals in this study. Note that for the frequently measured vitals we expect to see close to zero percent missingness, while for labs that are measured twice per day, we expect to see over 90 percent data missingness (100 - 2/24*100 = 91.7%).

Supplementary Table S 3: Summary of missingness percentage of the variables across four cohorts considered in this study.

Labs/Vitals	Hospital-A %missing	Hospital-B %missing	Hospital-C %missing	Hospital-D %missing
Heart rate	11.90	13.64	1.95	9.94
Pulse oximetry	13.10	15.61	2.04	17.90
Temperature	63.37	66.74	5.80	46.53
Systolic BP	36.79	15.38	2.25	10.69
Mean arterial pressure	37.00	16.41	2.02	11.57
Diastolic BP	36.80	15.39	2.26	10.69
Respiration rate	13.04	21.47	1.96	11.34
End tidal CO_2	88.36	93.62	90.51	97.32
Excess bicarbonate	96.99	99.81	93.12	95.63
Bicarbonate	97.00	99.86	91.34	99.68
Fraction of inspired Oxygen	80.41	97.85	41.66	90.98
pH	96.98	97.88	88.40	95.57
Partial pressure of CO_2 from arterial blood	97.00	98.87	79.46	95.63
Oxygen saturation from arterial blood	97.00	98.20	80.55	99.72
Aspartate transaminase	97.82	98.17	69.40	96.55
Blood Urea Nitrogen	92.44	94.38	84.22	92.76
Alkaline phosphatase	97.81	98.17	88.69	96.55
Calcium	92.54	93.07	83.99	92.72
Chloride	92.55	99.44	83.65	92.75
Creatinine	92.50	94.37	88.15	92.74
Bilirubin direct	99.26	99.77	95.07	96.55
Serum Glucose	92.48	78.32	42.25	82.45
Lactic acid	98.59	98.23	91.34	98.06
Magnesium	94.16	95.23	76.42	95.07
Phosphate	94.60	97.07	87.74	95.73
Potassium	92.01	92.37	43.51	92.68
Total Bilirubin	97.84	98.16	73.09	96.55
Troponin I	98.63	97.90	72.17	98.67
Hematocrit	92.45	94.19	43.68	92.44
Hemoglobin	92.44	94.14	89.73	89.69
Partial Thromboplastin Time	96.09	99.06	89.95	99.37
White Blood Cell count	92.45	94.69	89.08	92.82
Fibrinogen	99.40	99.51	99.81	99.55
Platelets	92.46	94.63	84.59	92.84

Supplementary Note: Exclusion Flowchart

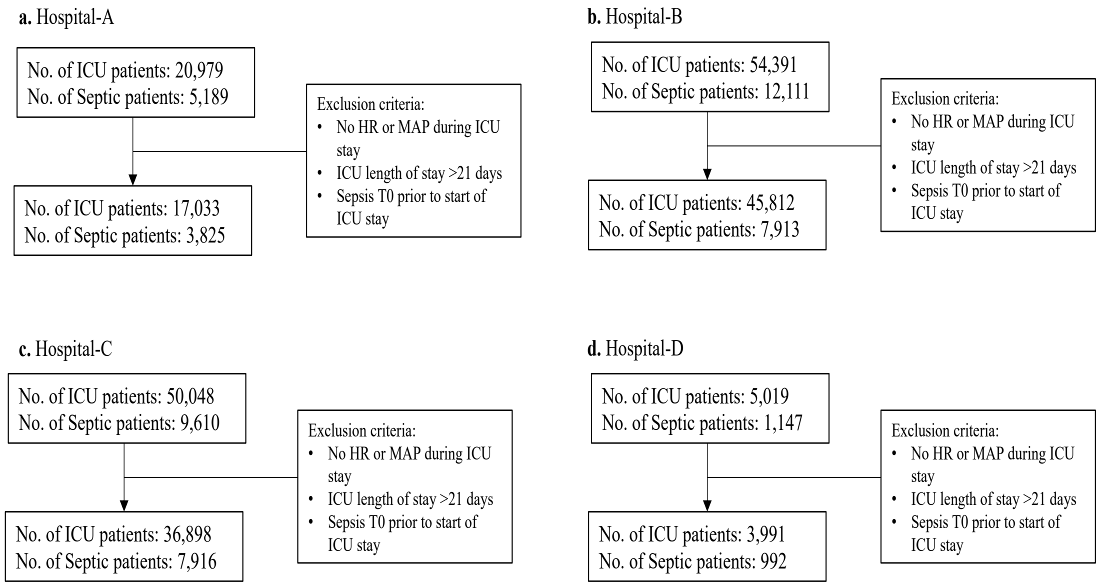

Supplementary Figure S3: Flowchart of population study across the four cohorts considered in this study.

Supplementary Note: Comparing the WUPERR performance with other baseline continual learning approaches

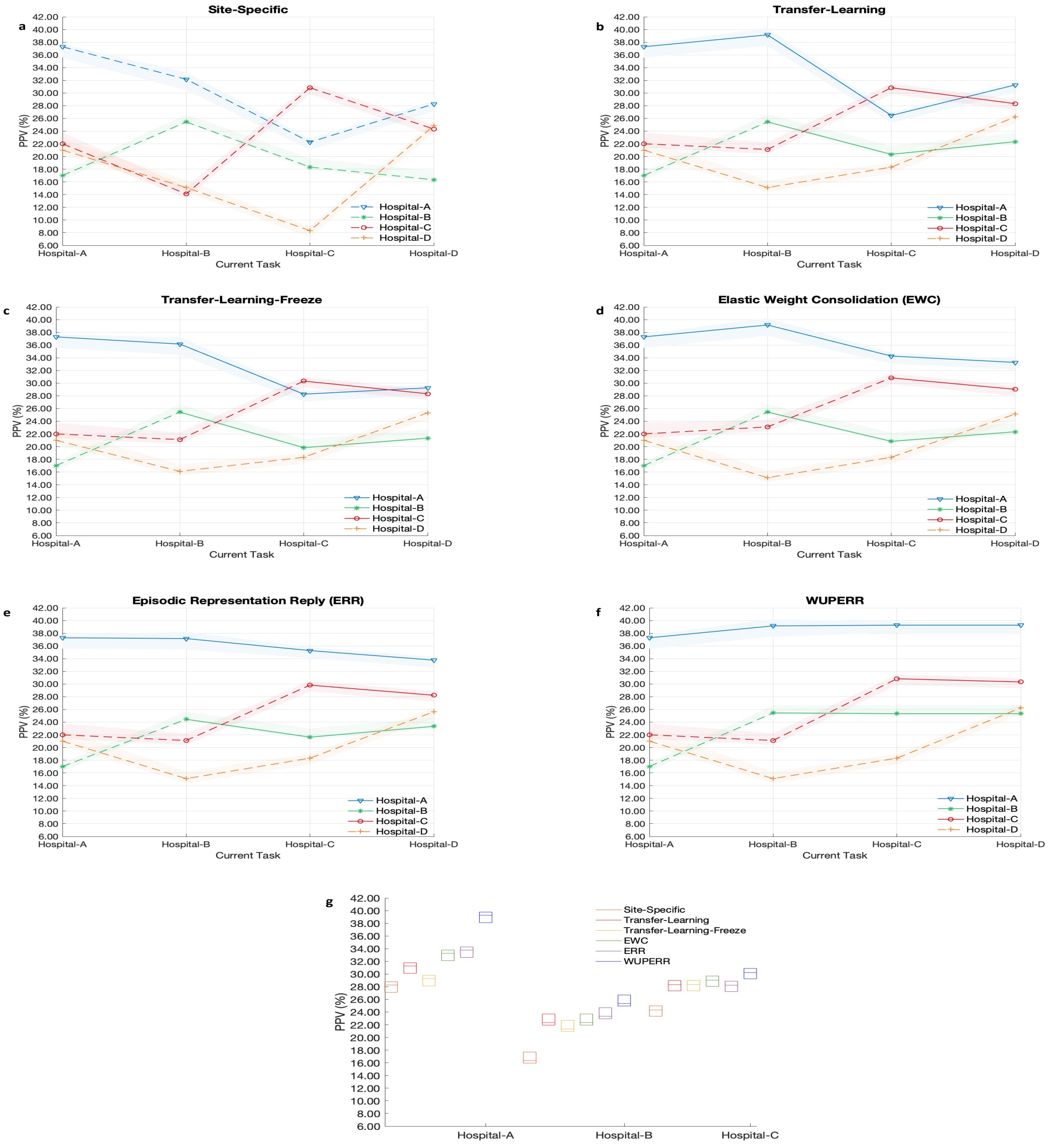

Supplementary Figure S4: Evaluation of continual learning models for early prediction of sepsis. Panel (a) illustrates the positive predictive value (PPV) of four separate models trained at each site separately on all the other sites. In all cases, PPV was calculated at a fixed threshold of 0.41, corresponding to 80% sensitivity at Hospital-A. For instance, the model trained at hospital-C (with PPV of 31%) performs poorly on hospital D (PPV of 24%). Panel (b) illustrates the model PPV on sequentially learning to predict the onset of Sepsis across four distinct hospitals using Transfer learning. Panels (c-f) shows the same for Transfer-Learning-Freeze, Elastic Weight Consolidation (EWC), Episodic Representation Replay (ERR), and the proposed WUPERR method, respectively. Panel (g) shows PPV values on Hospitals A-C after continual learning on all four hospitals with site-Specific (orange), Transfer learning (red), EWC (green), ERR (purple) and WUPERR (blue). Please refer to Fig. 2 of the main manuscript for an explanation of experimental setup and figure legends.

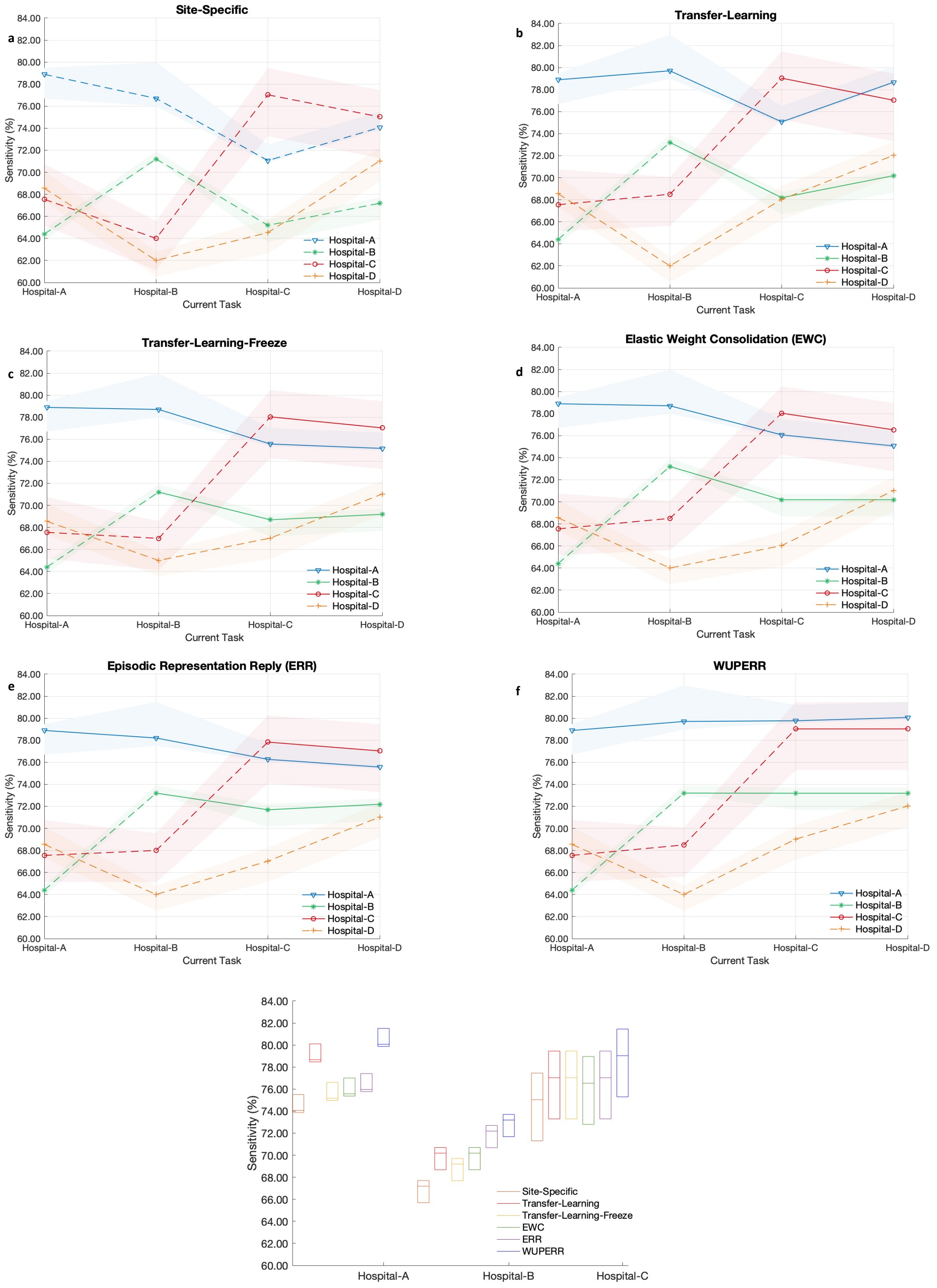

Supplementary Figure S5: Evaluation of continual learning models for early predicting of sepsis. Same as the previous figure but here we summarize the model sensitivity at the same fixed threshold of 0.41.

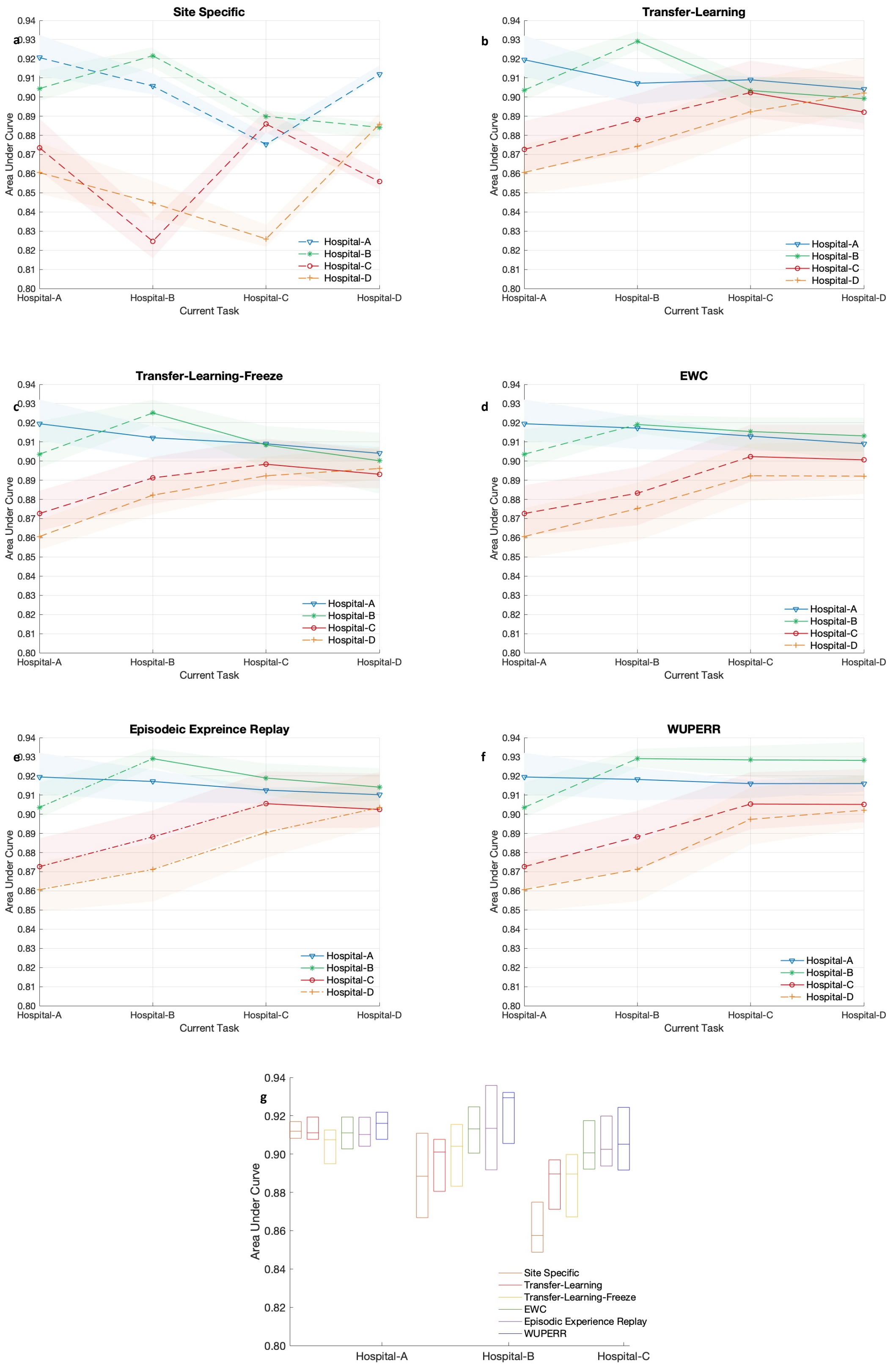

Supplementary Figure S6: Evaluation of continual learning models for early predicting of sepsis measured using Area Under the Curve (AUC) metric.

Supplementary Note: WUPERR consistently outperforms the baseline Transfer Learning irrespective to the order of tasks

Supplementary Figures S6-S11 illustrate the evaluation of the proposed WUPERR and baseline Transfer-Learning continual learning algorithms using PPV and Sensitivity metrics for early predicting of Sepsis when the ordering of hospitals are swapped.

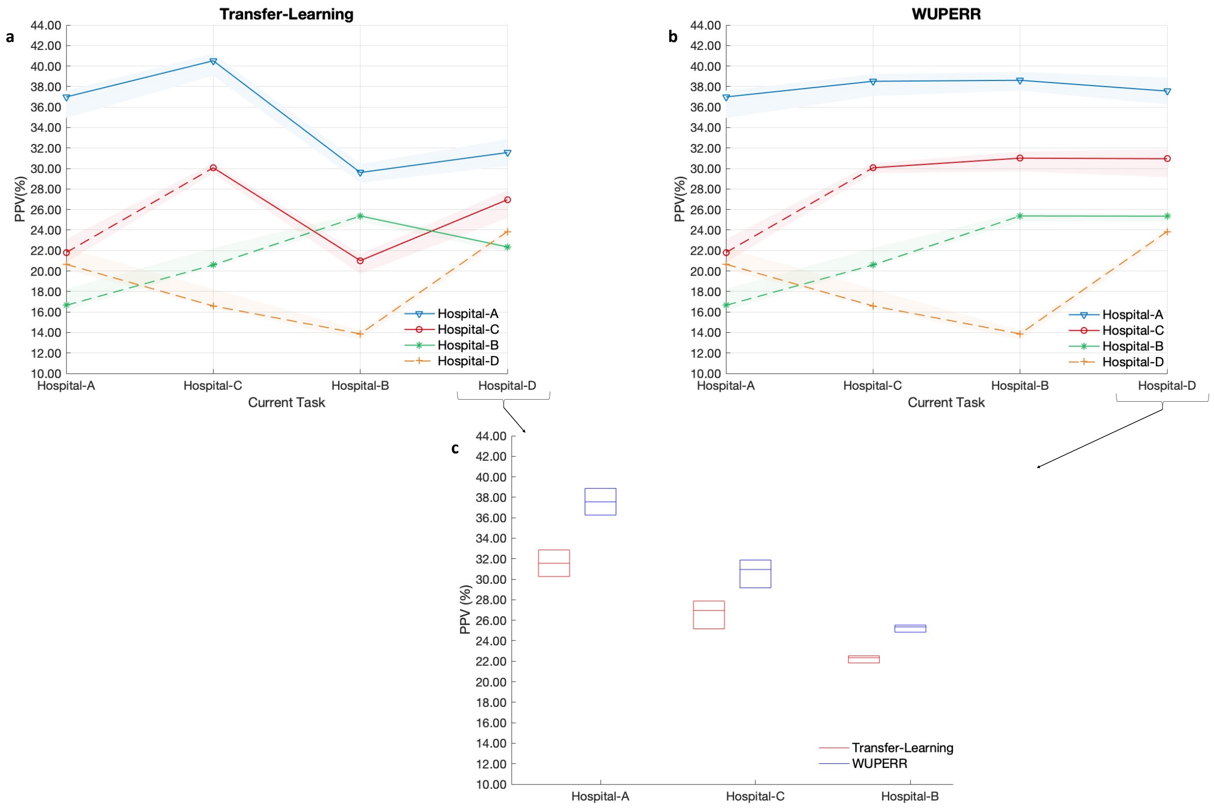

Supplementary Figure S7: Evaluation of continual learning models for early predicting of onset of Sepsis measured using PPV metric (tasks reordered as Hospital-A, Hospital-C, Hospital-B, and Hospital-D). Please refer to Fig. 2 of the main manuscript for an explanation of experimental setup and figure legends.

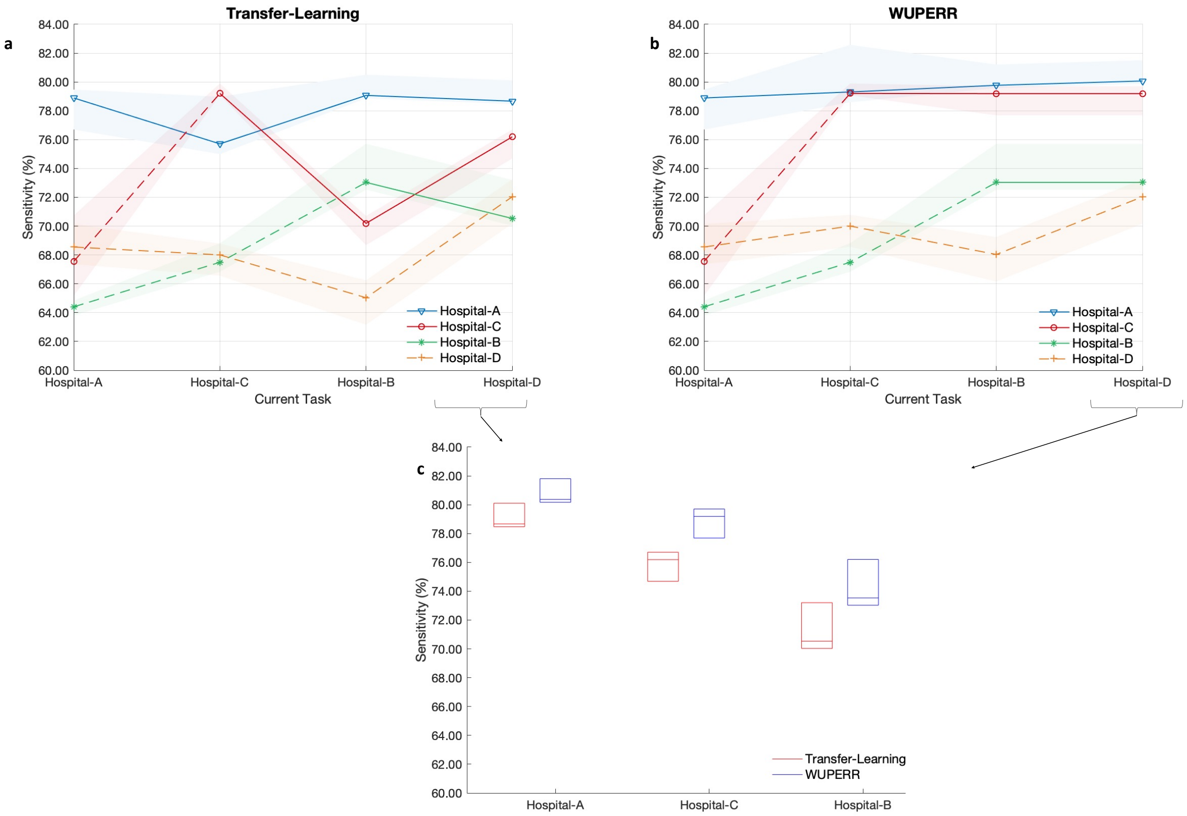

Supplementary Figure S8: Evaluation of continual learning models for early predicting of onset of Sepsis measured using Sensitivity metric (tasks reordered as Hospital-A, Hospital-C, Hospital-B, and Hospital-D). Please refer to Fig. 2 of the main manuscript for an explanation of experimental setup and figure legends.

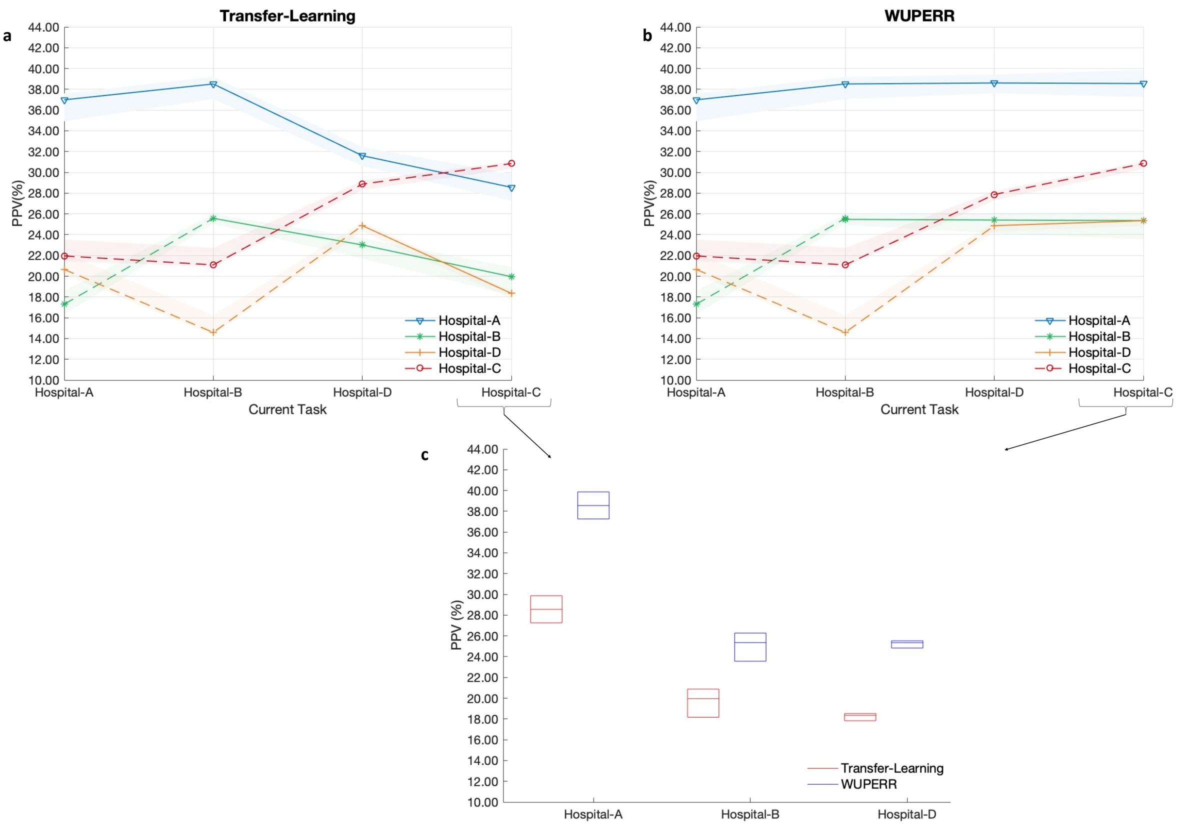

Supplementary Figure S9: Evaluation of continual learning models for early predicting of onset of Sepsis measured using PPV metric (tasks reordered as Hospital-A, Hospital-B, Hospital-D, and Hospital-C). Please refer to Fig. 2 of the main manuscript for an explanation of experimental setup and figure legends.

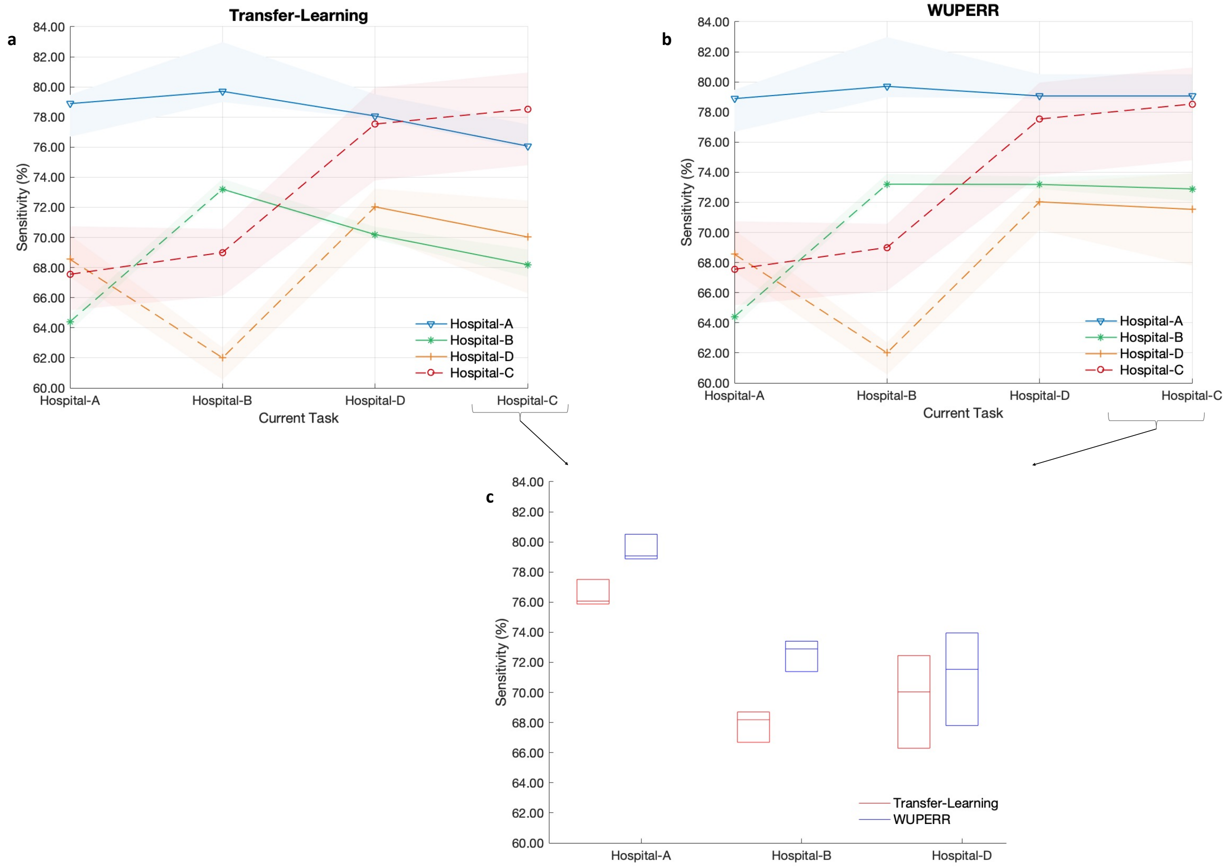

Supplementary Figure S 10: Evaluation of continual learning models for early predicting of onset of Sepsis measured using Sensitivity metric (tasks reordered as Hospital-A, Hospital-B, Hospital-D, and Hospital-C). Please refer to Fig. 2 of the main manuscript for an explanation of experimental setup and figure legends.

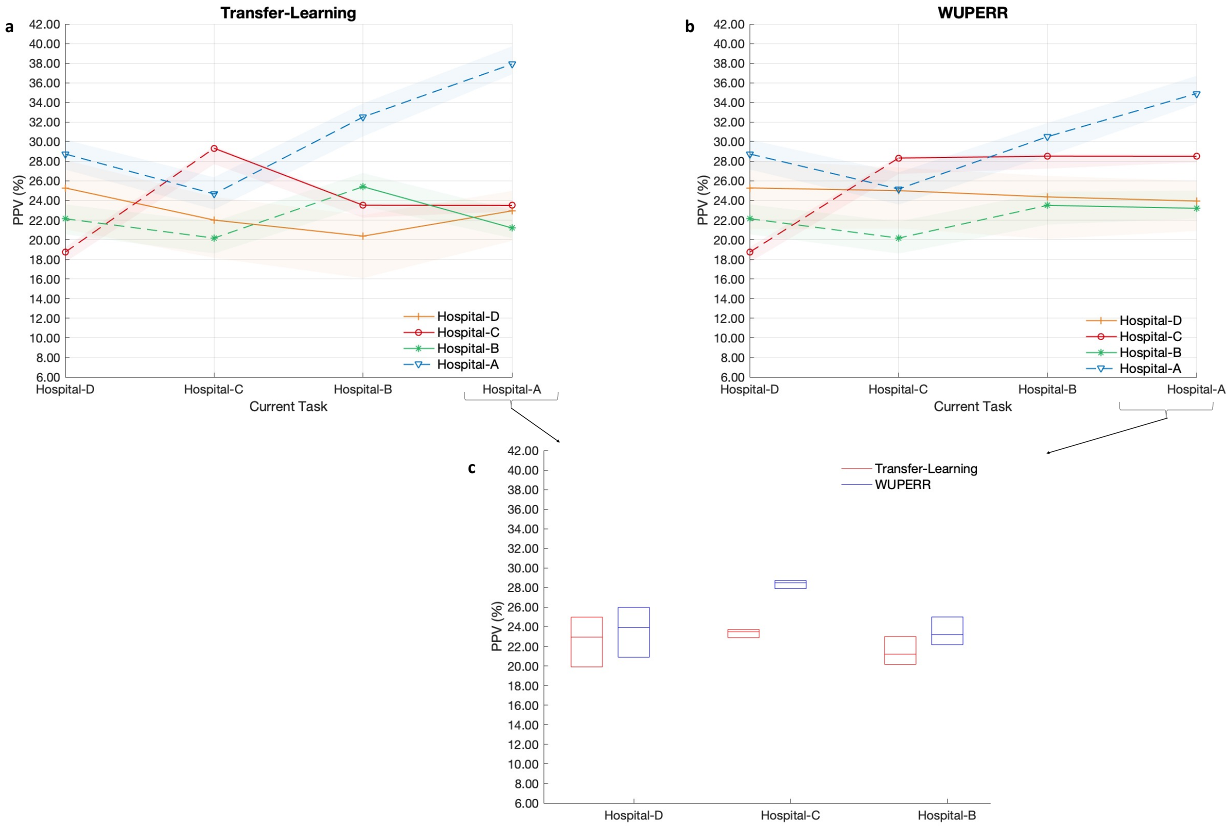

Supplementary Figure S11: Evaluation of continual learning models for early predicting of onset of Sepsis measured using PPV metric (tasks reordered as Hospital-D, Hospital-C, Hospital-B, and Hospital-A). Please refer to Fig. 2 of the main manuscript for an explanation of experimental setup and figure legends.

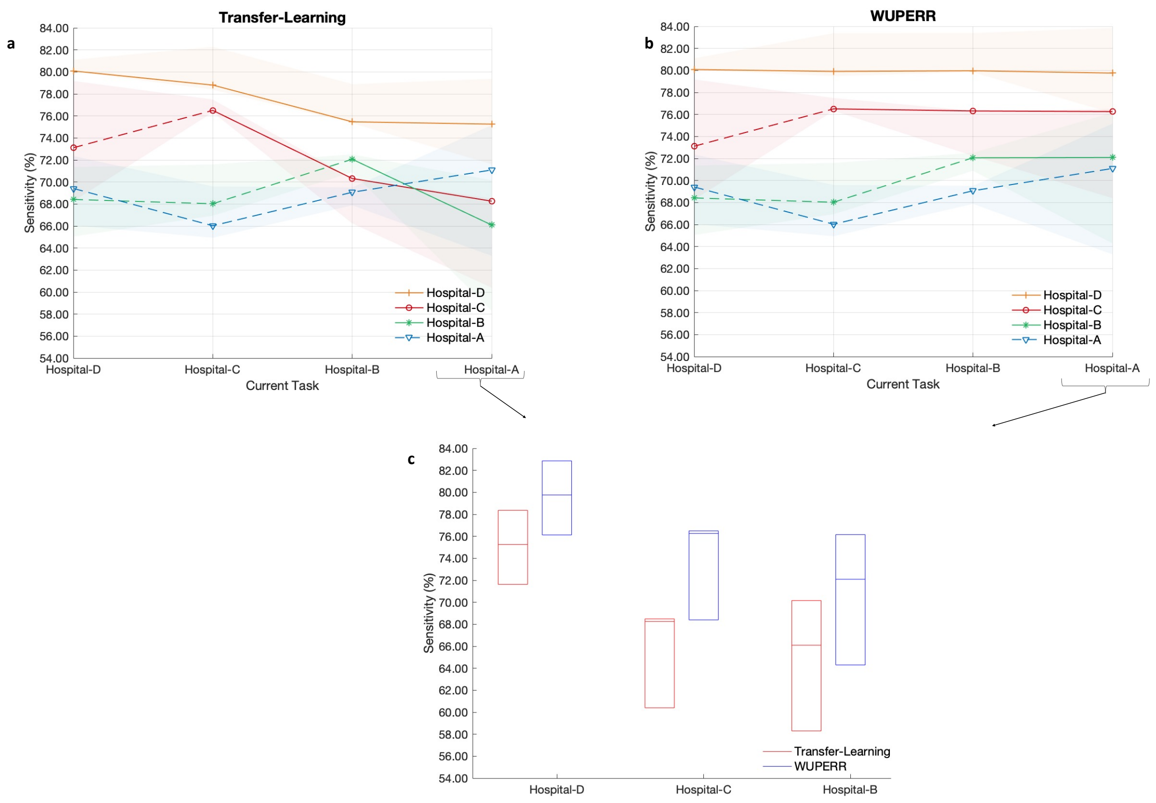

Supplementary Figure S 12: Evaluation of continual learning models for early predicting of onset of Sepsis measured using Sensitivity metric (tasks reordered as Hospital-D, Hospital-C, Hospital-B, and Hospital-A). Please refer to Fig. 2 of the main manuscript for an explanation of experimental setup and figure legends.

Supplementary Note: Deeper layers of Deep learning models are the source for forgetting

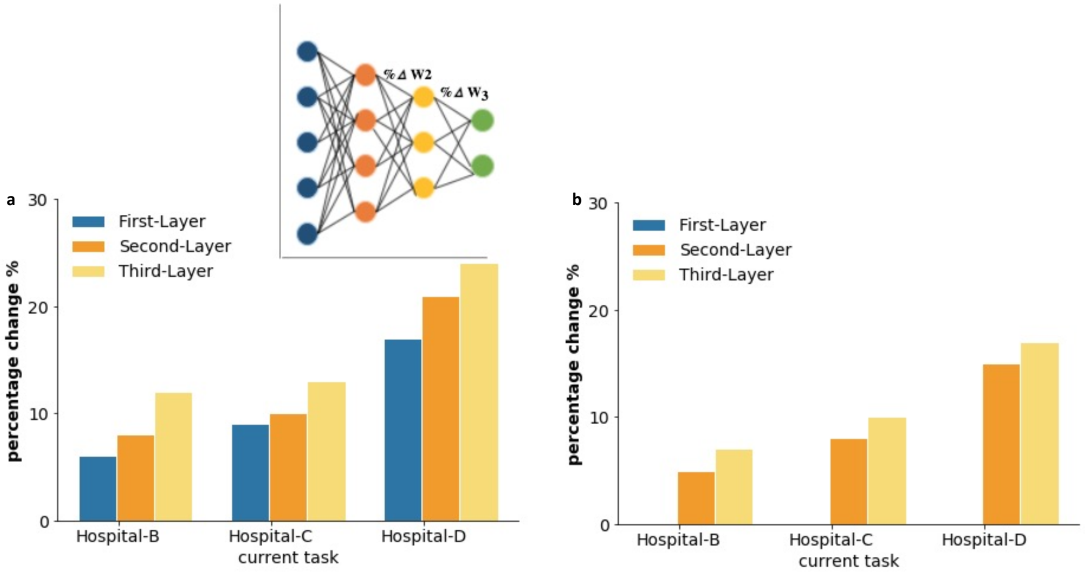

Supplementary Figure S 13: Layer-wise relative change in network weights. The bar plots represent the layer-wise percent change in the Frobenius norm of each weight matrice compared to the original weight matrix from Hospital A. The percent change for each layer are color coded, and the percent changes are calculated after training on each task (i.e., training on data from hospitals B, C, and D). The figure indicates that the higher layer weights change more significantly with learning of new tasks. Panel (a) show the percent changes when applying Transfer Learning, while panel (b) summarizes the results for the WUPERR algorithm. Note, since in WUPERR the weight matrix associated with the first layer is frozen, the associated percent change is always zero and thus has been omitted.
